# Ridge Alterations following Socket Preservation Using a Collagen Membrane in Dogs

**DOI:** 10.1155/2020/1487681

**Published:** 2020-03-03

**Authors:** Chengqi Lyu, Zhengwei Shao, Derong Zou, Jiayu Lu

**Affiliations:** Department of Stomatology, Shanghai Jiao Tong University Affiliated Sixth People's Hospital, Shanghai, China

## Abstract

**Background:**

The healing process following tooth extraction results in alveolar ridge resorption. The dimensional changes may complicate the subsequent implant procedure. Socket preservation using absorbable collagen membranes or a combination of membranes with calcium phosphate cement (CPC) particles might ensure that the alveolar ridge retains a suitable morphology for implant placement.

**Objective:**

To evaluate the quality and quantity of new bone regenerated after application of either collagen membranes alone covering the sockets or a combination of membranes with CPC particles added into the sockets in dogs. *Materials and Methods*. Six dogs were included in this study. The mandibular premolars were extracted. For each hemimandible, three premolar extraction sites were randomly assigned to one of the following treatments: a covering collagen membrane, CPC with a covering collagen membrane, and a socket left empty. Cone-beam computed tomography (CBCT) measurements, polyfluorochrome sequential labeling, and histological assessments were performed to investigate the healing ability and repair processes within a 6-month observation period.

**Results:**

Buccal bone height in the membrane group was significantly higher than that in the membrane+CPC and blank groups at 4 and 6 months after extraction. The mineral apposition rate over 2-4 months and the alizarin red-stained area in the membrane group were significantly higher than those in the other two groups. Histological analysis after 6 months of healing showed significantly higher amounts of newly formed bone in the membrane group than in the other groups.

**Conclusion:**

Extraction sites treated with collagen barrier membranes showed better protection than sites not covered with membranes. And the buccal bone wall of the socket was well preserved by collagen membrane without extra CPC materials. Socket preservation using absorbable membranes alone yielded better quality and quantity of regenerated bone inside the socket site.

## 1. Introduction

Dental implants supported prostheses represent one of the most optimal methods for oral rehabilitation because of their significantly ideal aesthetics, low failure rates, high masticatory efficiency, etc. [1]. Ideal functional and aesthetic prosthetic reconstruction following implant therapy requires sufficient alveolar bone volume in both vertical and horizontal dimensions [2]. However, physiological atrophy of the alveolar ridge occurs rapidly after tooth extraction and is primarily noted in the first 6 months postextraction [3]. The morphological changes in the extraction socket can be observed in the apical-coronal (vertical) and buccal-lingual (horizontal) dimensions [4]. In addition, during the process of recovery, food debris and the rapidly growing connective tissue can enter into the deep open wound and disturb bone regeneration [5]. Furthermore, the bundle bone of the buccal bone wall, which is a part of the periodontium, loses its function after tooth removal and starts undergoing resorption. Thus, buccal bone loss is more obvious than lingual bone loss, making it more difficult to achieve the aesthetic standards expected of dental implants [6]. The loss of alveolar bone volume also complicates implantation surgery by necessitating additional augmentative therapy or even making the placement of the implant impossible [7].

Socket preservation after tooth extraction has been regarded as an important step to ensure that the alveolar ridge retains a suitable morphology for an implant site. Bone substitute materials implanted into fresh sockets with a barrier membrane covering them could interfere with the bone resorption process and limit the atrophy of the alveolar ridge [8]. However, conflicting data have been reported regarding the outcome of using bone substitutes for preserving the extraction socket. Some reports have demonstrated that they cannot prevent resorption and that they lead to a reduction in the height of the buccal bone crest [9]. There is also a controversy regarding the quality of the bone augmented in the extraction socket. Histological observations have shown that the augmented bone mainly contains connective tissue and material particles after 6 to 9 months [10].

Barrier membranes have been shown to preserve alveolar ridges and provide beneficial results following tooth extraction in clinical trials [11]. The artificial membrane could seal off the socket for a healing period of up to several weeks. Extraction sockets covered by porcine-derived collagen membrane alone showed significantly lower vertical and horizontal bone changes, compared to spontaneous healing [12]. Moreover, calcium phosphate cement (CPC) may also be useful for increasing the height of the alveolar ridge [13]. However, the differences in socket preservation achieved by using the barrier membrane alone and the barrier membrane in combination with additional grafting material have not been fully investigated. Studies have also not clarified which approach provides regenerated bone with better quality and is more suitable for dental implants. In this study, we evaluated the effects of socket preservation using collagen membrane alone or in combination with CPC particles. Cone-beam computed tomography (CBCT) measurements, polyfluorochrome sequential labeling, and histological observations were used to assess the healing ability and repair processes associated with these two methods.

## 2. Materials and Methods

### 2.1. Animals

The animal selection, management, and experimental protocols were approved by the Animal Care and Experiment Committee of Shanghai Jiao Tong University Affiliated Sixth People's Hospital (Animal Welfare Ethics acceptance number: No.DWLL2017-0316) and complied with the ARRIVE guidelines [14]. A total of six healthy adult male beagles aged 18 months, each weighing 15.0 to 20.0 kg, were used in the study. All animals had a healthy, fully erupted permanent dentition. The dogs received standard food and water ad libitum.

### 2.2. Surgical Procedure

All animals were fasted for 48 hours before surgery but were allowed water ad libitum. General anesthesia was achieved by intravenous administration of pentobarbital sodium (30 mg/kg). After orotracheal intubation, the animals were monitored by a heart monitor during the entire course of the surgery.

**Figure 1:**
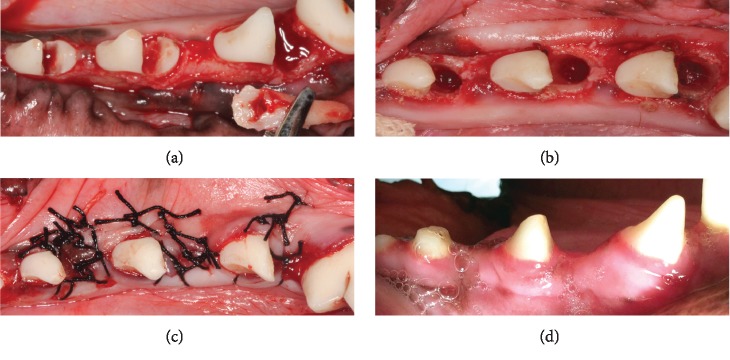
Surgical procedures for hemisectioning of the tooth. Teeth were hemisected (a), and the distal roots were extracted (b). The sockets were then sutured (c), and the extraction sockets recovered well (d) 6 months after surgery.

Buccal and lingual full-thickness flaps were made to expose the alveolar crests of the mandibular premolar regions on both sides. Three premolars (P2, P3, and P4) were hemisected on both sides using fissure burs (Figure 1(a)). The canals of all mesial roots were cleaned and filled with gutta-percha. The coronal parts of the pulp chambers were sealed with resin (Clearfil Core; Kuraray, Tokyo, Japan). The distal roots were carefully extracted using elevators and forceps (Figures 1(a) and 1(b)).

For each hemimandible, three premolar extraction sites were randomly assigned to one of the following treatments: (1) a covering double-layered collagen membrane (Bio-Gide, Geistlich Pharma AG, Wolhusen, Switzerland) (Bio-Gide group), (2) CPC (Rebone Biomaterials Co. Ltd., Shanghai, China) with a covering double-layered collagen membrane (Bio-Gide+CPC group), and (3) a socket left empty (blank group). To achieve primary healing, the buccal and lingual flaps were repositioned and sutured together using interrupted sutures with nonresorbable suture materials (Figure 1(c)). To prevent postoperative infections, the animals were given penicillin and kept on a soft diet for 7 days. Teeth were cleaned with a toothbrush and dentifriced three times per week for 4 weeks. The sutures were removed 2 weeks after the operation.

### 2.3. CBCT Measurements

The maxillas were scanned using the ProMax 3D CBCT (PLANMECA, Helsinki, Finland) immediately after surgery (M0). Three more CBCT scans were performed at 2 (M2), 4 (M4), and 6 months (M6) after surgery. A large-volume CBCT scan was performed in each mode with a rotation of 360 degrees. The voxel size was 0.2 mm, and the exposure factors were 90 kV and 14.0 mA. A series of axially sliced image data were exported to a personal computer in DICOM 3.0 format and reconstructed using the interactive setting on the Simplant software.

### 2.4. Vertical Measurements

**Figure 2:**
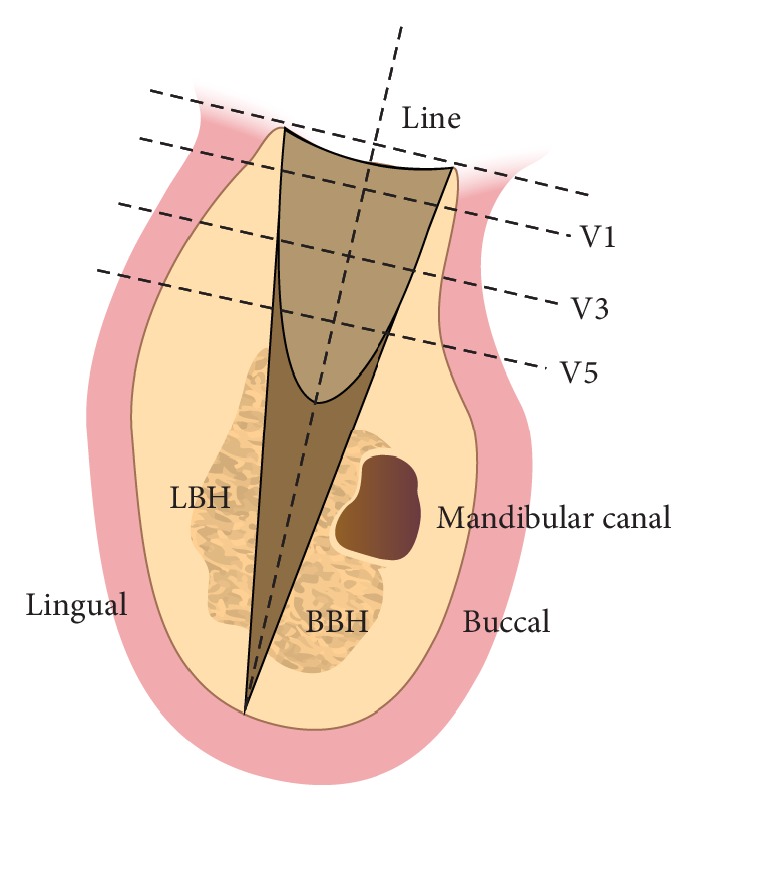
Schematic graph of vertical and horizontal CBCT measurements of the tooth extraction socket.

The vertical distances, i.e., the buccal and lingual bone height (BBH, LBH), were determined as follows (Figure 2). Reconstructed cross-sectional images were recorded from the central area of each extraction site as well as their corresponding alveolus in the buccolingual plane [15]. In the buccolingual plane, a line was placed from the long axis of the socket to the apical point of the jaw. Then, BBH and LBH were measured from the top of the buccal and lingual alveolar wall to the apical point. *△*BBH (M2, M4, or M6) and *△*LBH (M2, M4, or M6) were defined as BBH (M2, M4, or M6) − BBH (M0) and LBH (M2, M4, or M6) − LBH (M0), respectively. The difference between the buccal and lingual bone heights (BBH-LBH) was also calculated.

### 2.5. Horizontal Measurements

The horizontal distances between the buccal and lingual alveolar walls were measured by determining the total bone width (V1, V3, and V5). The distances were measured perpendicular to the line at 1, 3, and 5 mm underneath the top of the residual alveolar ridge.

### 2.6. Polyfluorochrome Sequential Labeling

Polyfluorochrome sequential labeling was performed to assess the new bone formation and mineralization processes in the extraction sockets. The beagles received tetracycline hydrochloride 25 mg/kg, alizarin red S 30 mg/kg, and calcein green 20 mg/kg (Sigma, Chemical Co, St Louis, MO, USA) subcutaneously at 2, 4, and 6 months after surgery, respectively.

### 2.7. Sample Preparation and Observation

The experiments were terminated 6 months after tooth extraction. The beagles were euthanized by intravital intracarotid perfusion under general anesthesia. Ten liters of physiologic NaCl solution were applied to the carotid artery under 120 mmHg pressure to wash out the blood from the vessels. The beagles were subsequently perfused with 10% neutral buffered formalin for internal fixation of the tissues. Then, the mandibles were immediately dissected, and the overlying soft tissues were scraped away. The segments containing the extraction sockets were block resected and fixed in 10% neutral buffered formalin for 4 days.

The segments were dehydrated using a series of ethanol solutions with increasing concentrations and embedded in polymethylmethacrylate (Sigma, Chemical Co, St Louis, MO, USA) for 14 days for polymerization. Samples were processed as follows: (1) 8 hours in 70% ethanol; (2) 8 hours in 80% ethanol; (3) 8 hours in 90% ethanol; (4) 8 hours in 95% ethanol; (5) 24 hours in 100% ethanol; (6) 8 hours in xylene; (7) 24 hours in xylene: penetration liquid (PMMA : dibutyl phthalate = 3 : 1) = 1 : 1 at 4°C; (8) 24 hours in embedding solution I (1% benzoyl peroxide (BPO) in penetration liquid) at 4°C; (9) 24 hours in embedding solution II (4% BPO in penetration liquid) at 4°C; and (10) replace the embedding solution II again, vacuum for 4 hours, and polymerize slowly at 37°C. Each specimen was cut and ground in the buccolingual direction along the long axis of the extraction socket using the Exact Cutting and Grinding Equipment (Exact Apparatebau, Norderstedt, Germany) to a thickness of 100 *μ*m. The sections were further ground by microgrinding and polishing to a thickness of 40 *μ*m for fluorescent labeling and histological observation.

Fluorescent labeling was observed under a confocal laser scanning microscope (LSM710; CarlZeiss, Germany). Fluorochrome staining images of the newly formed bone were stored digitally and then evaluated with an image analysis system (Image-Pro PlusTM 6.0; Media Cybernetics, USA) to explore bone formation and mineralization in the sockets. The mineral apposition rate (MAR, *μ*m/day) and the single fluorochrome stained area (%) were calculated according to the American Society for Bone and Mineral Research histomorphometry nomenclature committee's protocol/method [16].

The sections were further stained with Van Gieson's picric acid fuchsin to quantitatively evaluate the newly formed bone within the defect by using the Olympus light microscope (Olympus BX51; Tokyo, Japan), and the data were analyzed with an image analysis system (Image-Pro PlusTM 6.0, MD, USA). The percentage of the new bone area in the extraction socket was calculated by determining the ratio of the new bone area to the total socket area.

### 2.8. Statistical Analysis

Three premolar extraction sites were randomly assigned to one of the three treatments and at each side of the mandible. Measurements were made for each site, and a mean value was calculated for the two sites of the same group. Hence, the dog was used as a unit. All the data are shown as mean values ± standard deviation. The Shapiro–Wilk test was used to test the normality of the distribution. As all the measurements were normally distributed, one-way analysis of variance (ANOVA) with least significant difference (LSD) was applied to compare the differences between the three groups. All statistical analyses were performed using SPSS (SPSS 19.0, Chicago, IL, USA) statistical software packages. A two-tailed *p* value < 0.05 between the testing groups was considered to indicate statistical significance.

## 3. Results

### 3.1. Clinical Observations

All the extraction sockets recovered well after surgery. No adverse events such as infections or wound dehiscence occurred in relation to the surgical protocol in any of the animals during the entire observation period. Visual assessment of wound healing indicated that the local gingival tissue was in good condition in each group (Figure 1(d)).

### 3.2. CBCT Evaluation

**Figure 3:**
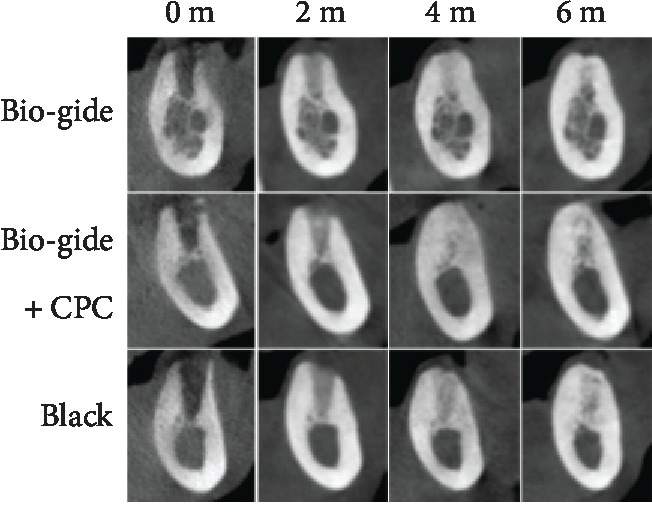
Representative CBCT images of extraction sockets at several time points after surgery.

CBCT images displayed the bone formation in the extraction sockets at several time points after surgery (Figure 3). As the healing period progressed, the sockets were filled with the newly formed bone. In the Bio-Gide+CPC and blank groups, the tops of the buccal alveolar wall were noticeably lower than the lingual tops. However, in the Bio-Gide group, the gaps between the tops were much smaller (Figure 3).

**Figure 4:**
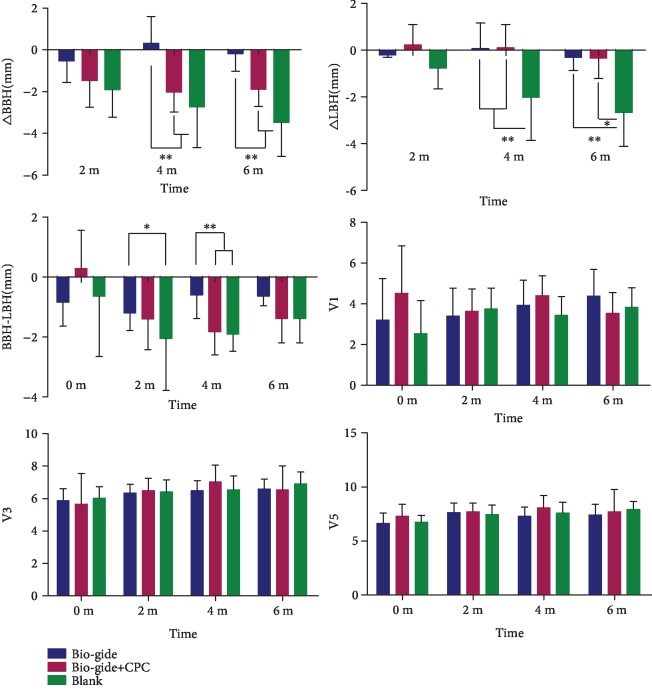
CBCT measurements of extraction sockets at several time points after surgery, including *△*BBH, *△*LBH, BBH-LBH, V1, V3, and V5. Mean values ± standard deviation. ^∗^*p* < 0.05; ^∗∗^*p* < 0.01.

Vertical measurements showed that the *△*BBH (M4) (0.31 ± 1.29 mm) and *△*BBH (M6) (−0.18 ± 0.85 mm) in the Bio-Gide group were significantly higher than those in the Bio-Gide+CPC and blank groups (Figure 4). Four months after the operation, the buccal alveolar wall bone height in the Bio-Gide+CPC and blank groups decreased more remarkably than that in the Bio-Gide group. The *△*LBH (M4) and *△*LBH (M6) values in the Bio-Gide (0.07 ± 1.08 and −0.31 ± 0.57 mm) and Bio-Gide+CPC (0.09 ± 1.01 and −0.34 ± 0.88 mm) groups were both higher than those in the blank group (−2.03 ± 1.84 and −2.67 ± 1.46 mm). In the blank group, both *△*BBH and *△*LBH were the lowest, indicating obvious reduction of both buccal and lingual bone height.

BBH-LBH was calculated to show the reduction in buccal bone height relative to lingual bone height. The values in the Bio-Gide group at 2 (−1.2 ± 0.58 mm) and 4 (−0.60 ± 0.78 mm) months after surgery were higher than those in the other groups. Thus, the relative reduction of BBH in the Bio-Gide group was the lowest. However, at 6 months postoperation, BBH-LBH was not significantly different among the three groups. This could be attributed to the greater reduction of LBH in the blank group, which also reduced the difference between BBH and LBH. There were no obvious differences in horizontal distances (V1, V3, and V5) among the three groups at all observation time points.

### 3.3. Fluorescent Labeling

**Figure 5:**
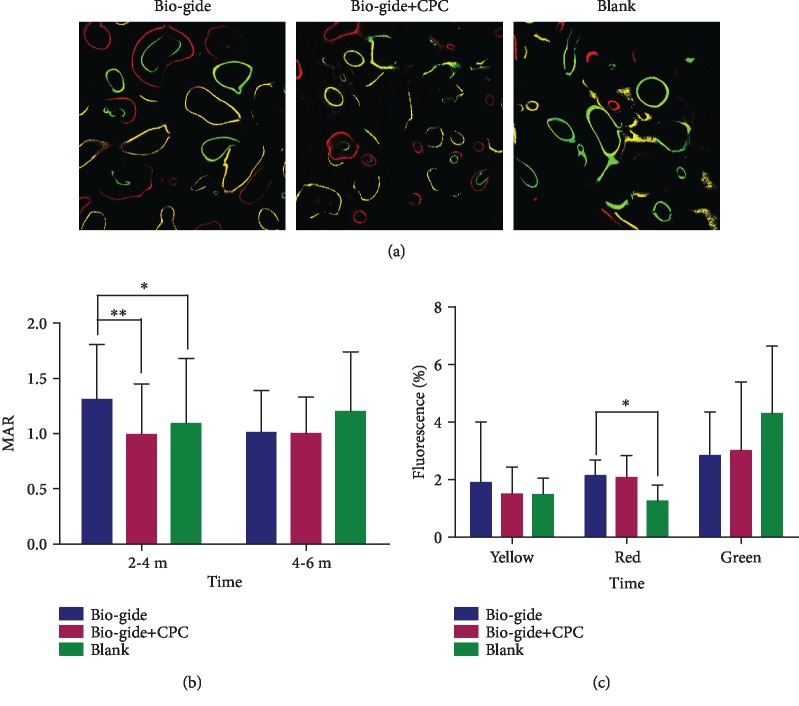
Fluorochrome labeling histomorphometric change analysis showed three-colored fluorescence rendering the newly formed bone in the extraction sockets (a). The yellow tetracycline labels appeared earliest at 2 months of surgery, followed by alizarin red and green calcein labels at 4 months and 6 months of treatment, respectively. Mineral apposition rate (MAR) was calculated after 2 to 4 months and 4 to 6 months of surgery (b). The single fluorochrome-stained area (%) was calculated at 2, 4, and 6 months after surgery (c). Original magnification, ×200. Mean values ± standard deviation. ^∗^*p* < 0.05; ^∗∗^*p* < 0.01.

Fluorescent labeling measurements were evaluated to assess bone histomorphometric indices among the different groups (Figure 5). The MAR value in the Bio-Gide group was significantly higher than those in the other two groups during 2-4 months (Figure 5(b)). Over 4-6 months after the operation, the histomorphometric indices in the three groups became similar. Although the medium-term (4 months) alizarin red-stained area (%) inside the extraction sockets in the Bio-Gide group was significantly greater than that in the blank group, no obvious intergroup differences were observed at 2 and 6 months after the operation, as shown by the yellow and green fluorescence-labeled areas (Figure 5(c)).

### 3.4. Histological Observations

**Figure 6:**
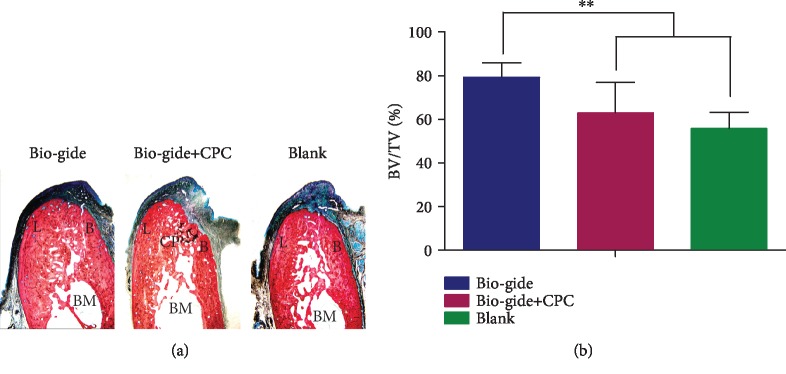
Histological observation of buccal-lingual sections of extraction sockets 6 months after extraction (a). The sockets were filled with a large amount of new bone, with BV/TV close to 80% and significantly higher than those in the other two groups (b). B: buccal bone wall; L: lingual bone wall; BM: bone marrow; CP: CPC particle. Van Gieson's stain; original magnification, ×12.5. Mean values ± standard deviation. ^∗∗^*p* < 0.01.

**Figure 7:**
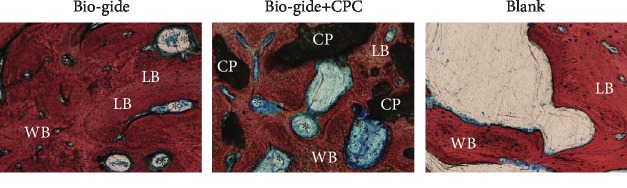
Higher magnification image of newly formed bone in the extraction sockets. LB: lamellar bone; WB: woven bone; CP: CPC particle; ^∗^blood vessel. Van Gieson's stain; original magnification, ×400.

After 6 months of healing, the buccal alveolar wall in the Bio-Gide group was significantly larger than that in the other two groups (Figure 6(a)). The extraction socket was bridged by newly formed mineralized bone composed of a large amount of lamellar bone and woven bone, which showed marrow space with a normal morphology and several blood vessels (Figure 7). No distinct border could be identified to distinguish the newly formed bone and the old bone in the buccal and lingual crest. The bone volume value (BV/TV, Bone Volume over Total Volume) of the Bio-Gide group was close to 80% and significantly higher than that of the other two groups (Figure 6(b)). No Bio-Gide membranes and surface invaginations can be seen in the sockets. In the Bio-Gide+CPC group, the remaining CPC material particles were found to contact intimately with the new bone in the socket (Figure 7). A substantial number of blood vessels were observed in the socket and even near the CPC particles. There are some depressions on the surface of the alveolar bone close to the remaining particles (Figure 6(a)). In the untreated blank group, the newly formed bone was obvious at the apical region of the defect but sparse in the socket.

## 4. Discussion

Considering the growing clinical demand for dental implant procedures, this study assessed the promotion of guided bone regeneration by using a barrier membrane alone and a membrane with grafting material. The quality and quantity of the regenerated bone formed in these two methods were investigated to define an adequate implant site.

After natural healing for 6 months, the height of the buccal bone wall of the tooth socket reduces remarkably by about 4 mm [17]. In this study, the sockets covered with Bio-Gide membranes alone showed significantly higher BBH than the Bio-Gide+CPC and blank groups at 4 and 6 months after extraction. With respect to LBH, both Bio-Gide and Bio-Gide+CPC groups showed significantly higher values than that in the blank group. The *△*BBH values in the Bio-Gide group were close to 0 mm at 6 months after extraction (*△*BBH, −0.18 ± 0.85 mm), suggesting that Bio-Gide collagen membrane almost completely preserved the buccal alveolar ridge around tooth sockets. However, another clinical randomized controlled study, using the same ridge preservation protocol, shows that the vertical alveolar height decreased 0.55 ± 0.11 mm at the extraction sockets treated with collagen membrane alone [12]. This difference in the vertical bone changes may be due to the different rates of the new bone apposition between human and animals [18]. In histological assessments conducted after 6 months of healing, the amount of the newly formed bone generated by the Bio-Gide membrane alone was significantly higher than that in the other two groups. The result was similar to that reported by Carmagnola et al. [10], who noted that collagen membrane-treated sockets in humans showed higher amounts of lamellar and woven bone compared to grafting material-treated sockets. Moreover, although horizontal ridge evaluations did not show any significant intergroup differences (Figure 4), which was not in agreement with the findings of previous studies, this finding is similar to the studies that used hemisection procedures [19, 20]. A possible reason for the discrepancy could be that the presence of another half root adjacent to the extraction site helped maintain the width of the socket.

Barrier membranes are widely accepted for used in guided tissue regeneration (GTR) in periodontal defects. The blockage of epithelial migration into bone defects by the membrane enhances bone regeneration by ensuring local concentration of osteoprogenitor cells and biological growth factors in the wound [21]. This advantage also decelerates the dimensional atrophy of the alveolar ridge after tooth extraction [12].

Geistlich Bio-Gide is a resorbable bilayer membrane obtained from the natural collagen of pigs without further cross-linking or chemical additives. The dense surface prevents the ingrowth of fibrous tissue into the bone defect, while the porosity of the membrane allows for the ingrowth of bone-forming cells [22]. Thus, the membrane can play a role in stabilizing the blood clot, maintaining space for bone regeneration in the socket, and protecting the wound from mechanical disruption and saliva contamination. Collagen barriers also have some specific physicochemical properties, including hemostatic activity that allows wound stabilization, chemotactic effects over gingival fibroblasts, and permeability that allows nutrient transfer [23]. Considering the ease of manipulation, decreased risk of flap dehiscence, and low immunogenicity associated with these membranes, they are the ideal barrier materials for socket preservation [24].

An important concern related to the use of bioabsorbable membranes is the speed at which resorption takes place. It is recommended that membranes remain in place for at least 4 to 6 weeks in order to achieve good regenerative results. In a previous study, residual membrane material of the double-layered collagen membrane was found at 3 months after tooth extraction, indicating a long-lasting barrier function [25]. The delayed resorption time could ensure an elongated barrier function and promote further new bone formation in the extraction socket [26, 27]. The MAR value at 2-4 months and the alizarin red-stained area (%) in the Bio-Gide group was significantly higher than the corresponding values in the other two groups, indicating that the elongated barrier function could be beneficial for improving the bone mineralization speed in the middle stage of healing.

Bone augmentation materials can be applied to the extraction sites to support the membrane from collapsing and maintain the dimensions of the alveolar ridge by enhancing new bone formation through osteoinduction and/or osteoconduction. Intrasocket grafts cannot achieve complete ridge preservation but can reduce the amount of resorption in comparison with spontaneous healing [28]. In this study, when the Bio-Gide membrane was combined with CPC to treat the socket, the preserved buccal and lingual bone walls were 2.59 mm and 2.33 mm, respectively, higher than that in the blank group at 6 months after tooth extraction. While the height of the preserved lingual bone wall was almost the same in the Bio-Gide+CPC and Bio-Gide groups, the corresponding value for the buccal bone wall in the Bio-Gide+CPC group was lower than that in the Bio-Gide group, indicating that collagen membrane alone may offer better protection of the buccal bone wall postextraction.

The use of grafting materials in fresh extraction sockets has been questioned because they could interfere with the normal healing process in the sockets in which oral implants are to be inserted. The grafting materials could have negative effects on revascularization [9, 29]. The reduction in vascular supply caused by intrasocket grafts may cause slower resorption of the alveolar ridge, because greater vascular supply could lead to faster resorption [30]. In the natural healing process, the socket is first occupied by a coagulum, which is successively replaced by granulation tissue, provisional connective tissue and woven bone, and finally lamellar bone and marrow [30]. The first signs of remodeling are seen with a vascular network and osteoid within a week, and even after 2 months, bone formation is incomplete and remodeling is in progress [31]. However, the CPC material-grafted sites exhibit a delayed healing pattern. Before a new bone can regenerate in the augmented site, the biomaterial has to undergo a “surface cleaning” that is associated with the presence of TRAP-positive multinucleated cells, i.e., osteoclasts, and thereby prepare enough space for deposition of the newly formed bone [32, 33]. Macrophages involve in the local chronic inflammation intimately after implantation of biomaterials. Collagen membranes are often designed to minimize foreign body reactions related to macrophages [27, 34]. Because of its natural structure, Bio-Gide collagen membrane is degraded without specific inflammation and thus is biocompatible [35]. However, recent research found that macrophages play a critical role during bone regeneration based on their polarization into both proinflammatory and anti-inflammatory phenotypes. Immune-responsive collagen membranes may be an innovative strategy for improving guided bone regeneration surgery [36, 37].

Biodegradation is directly influenced by the material properties and the size of the inserted particles. The porous CPC grafting material used in this study showed good biocompatibility and bioactivity [38]. The average pore size of the CPC scaffold was 300–500 mm, similar to the human cancellous bone. Since they are self-setting and resorbable under physiological conditions, the scaffolds are involved in tissue metabolism and could promote bone tissue growth after implantation in vivo. In our previous report, the materials showed complete contact with body fluids, dissolved, and collapsed quickly [39]. Three months later, the collapsed particles could be seen surrounded by multinucleated giant cells, and a new bone formation had not fully completed. The multinucleated giant cells are considered to play an important role to the resorption of implanted CPC particles and the balance of bone reconstruction. Thus, in the Bio-Gide+CPC-treated sites, there was less mineralized bone and a substantial amount of blood vessels and CPC particles remained in the extraction socket after 6 months of healing. In addition to the resorption of the graft and delayed regeneration of the new bone, a substantial vascular supply had been generated, indicating further resorption of the graft and alveolar ridge. Thus, a complete resorption of the implanted CPC granules and termination of bone regeneration would take a long time.

## 5. Conclusion

Within the limitations of the present animal study, considering both the dimensions and amount of newly formed bone in the extraction sockets, sites treated with a collagen barrier membrane showed better protection than sites not treated with membranes. In particular, the buccal bone wall of the socket was well preserved by Bio-Gide resorbable collagen membrane without extra CPC materials. The combination of Bio-Gide membrane and CPC particles could also reduce the amount of alveolar atrophy compared with that observed during spontaneous healing. Thus, preservation procedures employing absorbable membranes could ensure better quality and quantity of the regenerated bone at the socket site, which would be more suitable for dental implant application. On the other hand, the effect of collagen membrane on bone preservation for large postextraction defects remains to be explored.

## Data Availability

The data used to support the findings of this study are available from the corresponding author upon request.

## References

[1] Srinivasan M., Meyer S., Mombelli A., Muller F. (2017). Dental implants in the elderly population: a systematic review and meta-analysis. *Clinical Oral Implants Research*.

[2] Sanz M., Vignoletti F. (2015). Key aspects on the use of bone substitutes for bone regeneration of edentulous ridges. *Dental Materials*.

[3] Van der Weijden F., Dell'Acqua F., Slot D. E. (2009). Alveolar bone dimensional changes of post-extraction sockets in humans: a systematic review. *Journal of Clinical Periodontology*.

[4] Osorio L. B., de Menezes L. M., Assaf J. H., Soares A. V., da Veiga M. L., Stuani M. B. (2016). Post-extraction evaluation of sockets with one plate loss – a microtomographic and histological study. *Clinical Oral Implants Research*.

[5] Farina R., Trombelli L. (2011). Wound healing of extraction sockets. *Endodontic Topics*.

[6] Chappuis V., Araujo M. G., Buser D. (2017). Clinical relevance of dimensional bone and soft tissue alterations post-extraction in esthetic sites. *Periodontology 2000*.

[7] Milinkovic I., Cordaro L. (2014). Are there specific indications for the different alveolar bone augmentation procedures for implant placement? A systematic review. *International Journal of Oral and Maxillofacial Surgery*.

[8] Sheikh Z., Qureshi J., Alshahrani A. M. (2017). Collagen based barrier membranes for periodontal guided bone regeneration applications. *Odontology*.

[9] Morjaria K. R., Wilson R., Palmer R. M. (2014). Bone healing after tooth extraction with or without an intervention: a systematic review of randomized controlled trials. *Clinical Implant Dentistry and Related Research*.

[10] Carmagnola D., Adriaens P., Berglundh T. (2003). Healing of human extraction sockets filled with Bio-Oss. *Clinical Oral Implants Research*.

[11] Masaki C., Nakamoto T., Mukaibo T., Kondo Y., Hosokawa R. (2015). Strategies for alveolar ridge reconstruction and preservation for implant therapy. *Journal of Prosthodontic Research*.

[12] Guarnieri R., Stefanelli L., De Angelis F., Mencio F., Pompa G., Di Carlo S. (2017). Extraction socket preservation using porcine-derived collagen membrane alone or associated with porcine-derived bone. Clinical results of randomized controlled study. *Journal of Oral and Maxillofacial Research*.

[13] Sugawara A., Fujikawa K., Kusama K. (2002). Histopathologic reaction of a calcium phosphate cement for alveolar ridge augmentation. *Journal of Biomedical Materials Research*.

[14] Kilkenny C., Browne W. J., Cuthill I. C., Emerson M., Altman D. G. (2010). Improving bioscience research reporting: the ARRIVE guidelines for reporting animal research. *PLoS Biology*.

[15] Rothamel D., Schwarz F., Herten M. (2008). Dimensional ridge alterations following socket preservation using a nanocrystalline hydroxyapatite paste. A histomorphometrical study in dogs. *International Journal of Oral and Maxillofacial Surgery*.

[16] Parfitt A. M., Drezner M. K., Glorieux F. H. (1987). Bone histomorphometry: standardization of nomenclature, symbols, and units: report of the ASBMR Histomorphometry Nomenclature Committee. *Journal of Bone and Mineral Research*.

[17] Schropp L., Wenzel A., Kostopoulos L., Karring T. (2003). Bone healing and soft tissue contour changes following single-tooth extraction: a clinical and radiographic 12-month prospective study. *The International Journal of Periodontics & Restorative Dentistry*.

[18] Botticelli D., Lang N. P. (2017). Dynamics of osseointegration in various human and animal models - a comparative analysis. *Clinical Oral Implants Research*.

[19] Fickl S., Schneider D., Zuhr O. (2009). Dimensional changes of the ridge contour after socket preservation and buccal overbuilding: an animal study. *Journal of Clinical Periodontology*.

[20] Fickl S., Zuhr O., Wachtel H., Kebschull M., Hurzeler M. B. (2009). Hard tissue alterations after socket preservation with additional buccal overbuilding: a study in the beagle dog. *Journal of Clinical Periodontology*.

[21] Elgali I., Omar O., Dahlin C., Thomsen P. (2017). Guided bone regeneration: materials and biological mechanisms revisited. *European Journal of Oral Sciences*.

[22] Kunert-Keil C., Gredes T., Heinemann F., Dominiak M., Botzenhart U., Gedrange T. (2015). Socket augmentation using a commercial collagen-based product — an animal study in pigs. *Materials Science and Engineering: C*.

[23] Caballe-Serrano J., Munar-Frau A., Delgado L., Perez R., Hernandez-Alfaro F. (2019). Physicochemical characterization of barrier membranes for bone regeneration. *Journal of the Mechanical Behavior of Biomedical Materials*.

[24] Roman A., Cioban C., Stratul S. I. (2015). Ridge preservation using a new 3D collagen matrix: a preclinical study. *Clinical Oral Investigations*.

[25] Ivanovic A., Bosshardt D. D., Mihatovic I., Schwarz F., Gruber R., Sculean A. (2014). Effect of pulverized natural bone mineral on regeneration of three-wall intrabony defects. A preclinical study. *Clinical Oral Investigations*.

[26] Donos N., Bosshardt D., Lang N. (2005). Bone formation by enamel matrix proteins and xenografts: an experimental study in the rat ramus. *Clinical Oral Implants Research*.

[27] Chu C., Deng J., Sun X., Qu Y., Man Y. (2017). Collagen membrane and immune response in guided bone regeneration: recent progress and perspectives. *Tissue Engineering Part B: Reviews*.

[28] Festa V. M., Addabbo F., Laino L., Femiano F., Rullo R. (2013). Porcine-derived xenograft combined with a soft cortical membrane versus extraction alone for implant site development: a clinical study in humans. *Clinical Implant Dentistry and Related Research*.

[29] Omara M., Abdelwahed N., Ahmed M., Hindy M. (2016). Simultaneous implant placement with ridge augmentation using an autogenous bone ring transplant. *International Journal of Oral and Maxillofacial Surgery*.

[30] Irinakis T. (2006). Rationale for socket preservation after extraction of a single-rooted tooth when planning for future implant placement. *Journal of the Canadian Dental Association*.

[31] Bagoff R., Mamidwar S., Chesnoiu-Matei I., Ricci J. L., Alexander H., Tovar N. M. (2013). Socket preservation and sinus augmentation using a medical grade calcium sulfate hemihydrate and mineralized irradiated cancellous bone allograft composite. *Journal of Oral Implantology*.

[32] Araujo M. G., Liljenberg B., Lindhe J. (2010). Dynamics of Bio-Oss® Collagen incorporation in fresh extraction wounds: an experimental study in the dog. *Clinical Oral Implants Research*.

[33] Jensen S. S., Broggini N., Hjorting-Hansen E., Schenk R., Buser D. (2006). Bone healing and graft resorption of autograft, anorganic bovine bone and beta-tricalcium phosphate. A histologic and histomorphometric study in the mandibles of minipigs. *Clinical Oral Implants Research*.

[34] Chu C., Liu L., Rung S. (2020). Modulation of foreign body reaction and macrophage phenotypes concerning microenvironment. *Journal of Biomedical Materials Research. Part A*.

[35] Rothamel D., Schwarz F., Sager M., Herten M., Sculean A., Becker J. (2005). Biodegradation of differently cross-linked collagen membranes: an experimental study in the rat. *Clinical Oral Implants Research*.

[36] Chu C., Liu L., Wang Y. (2018). Macrophage phenotype in the epigallocatechin-3-gallate (EGCG)-modified collagen determines foreign body reaction. *Journal of Tissue Engineering and Regenerative Medicine*.

[37] Chu C., Wang Y., Wang Y. (2019). Evaluation of epigallocatechin-3-gallate (EGCG) modified collagen in guided bone regeneration (GBR) surgery and modulation of macrophage phenotype. *Materials Science and Engineering: C*.

[38] Shih T. C., Teng N. C., Wang P. D. (2013). In vivo evaluation of resorbable bone graft substitutes in beagles: histological properties. *Journal of Biomedical Materials Research. Part A*.

[39] Zou D., Guo L., Lu J. (2012). Engineering of bone using porous calcium phosphate cement and bone marrow stromal cells for maxillary sinus augmentation with simultaneous implant placement in goats. *Tissue Engineering Part A*.

